# Jump Across the Gap! A New Type of Colour Spreading Illusion

**DOI:** 10.1177/2041669518819511

**Published:** 2018-12-26

**Authors:** Kunihiro Hasegawa, Shin'ya Takahashi

**Affiliations:** National Institute of Advanced Industrial Science and Technology, Japan; Tokai Gakuen University, Japan

**Keywords:** colour, colour assimilation, colour spreading, contour capturing, contours/surfaces, contrast induction, filling-in, lightness/brightness

## Abstract

The present article reports a new illusory colour phenomenon. There have been previous reports of illusory colours that spread to an area in contact with a coloured object (e.g., neon-like spreading). However, according to our informal observations as well as the experiments reported here, illusory colour spreads even if the coloured and noncoloured areas are separated by a gap. The contour-based perception account as well as the interaction of some components of surround suppression in the visual cortex were discussed as possibilities to account for the present illusion. The effect reported in the present study may provide suggestions for further understanding of colour perception.

Colour perception often depends on the surrounding environment. For example, the background of the coloured parts of lines appears as a coloured surface when parts of noncoloured lines on a noncoloured background are coloured, referred to as neon-like spreading (Prandtl, 1927; van Tuijl, 1975). In another example, the colour of a thin chromatic contour spreads to a neighbouring nonchromatic surface, referred to as the watercolour illusion (Pinna, Brelstaff, & Spillmann, 2001). These colour spreading illusions provide some evidence of how the colour perception system judges surface colour.

The present study reports a new type of colour spreading illusion: The colour of an object spreads to a nearby object but not to an interspersed (noncoloured) area (Figure 1(a)). Our informal observations indicate that the illusory colour appears to grow progressively stronger when one has been staring at an image for a while. The geometric features of the coloured object(s) and the noncoloured object(s) need not be identical (Figure 1(b)), and the objects need not be closed (Figure 1(c) and (d)). The investigation of the commonalities and differences between the present and the previously identified colour spreading illusions may provide further insight into the mechanisms underlying colour perception.

**Figure 1.**
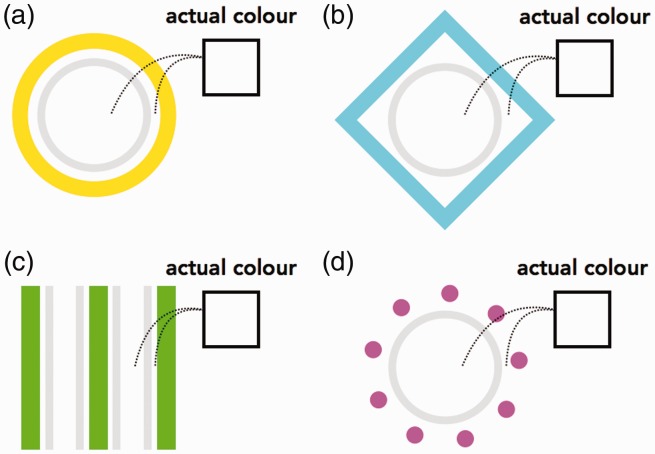
Examples of the newly identified illusion. Please continue to stare at the image for several seconds. The inside of the light grey contour appears to be faintly coloured.

The characteristic feature of this new spreading illusion is the presence of a “gap.” Previous colour spreading illusions have been reported, including the neon-like spreading, the watercolour effect, and the filling-in afterimage (van Lier, Vergeer, & Anstis, 2009). In these illusions, the illusory colour in the effect spreads to an area in contact or overlapping with the actual coloured area (Figure 2(a)). However, in the present newly identified illusion, the coloured area and the area to which the colour spreads are separated by a gap (Figure 2(b)). It appears that the contour captures the illusory colour.

**Figure 2.**
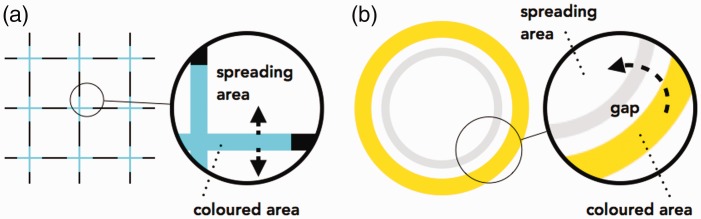
Spatial arrangement of the areas with actual and illusory colours in (a) the neon-like spreading and (b) the new illusion presented here.

To capture the illusory colour, the lightness contrast between the contour and the background cannot be too high (Figure 3). When the contrast of these was low, the illusory colour captured inside the contour (Figure 3(b) and (c)). However, the illusory colour became weak or disappeared when the contrast of these became too high (Figure 3(a) and (d)). In addition, the direction of the effect was independent of the brightness polarity of the contours. The yellowish surface is perceived within the contour of the inner circle when the contour is either lighter or darker than background (Figure 3(b) and (c)). Furthermore, the whiteness (or brightness) of the gap between the two objects appears to be enhanced compared with the background.

**Figure 3.**
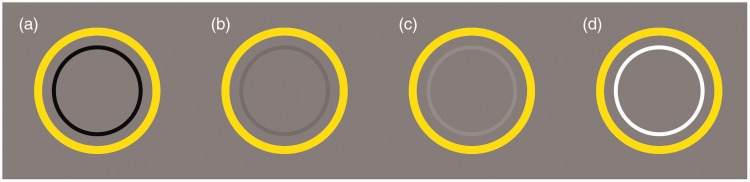
The relationship of the contrast between the background and the contour of the inner circle. The background is 50% grey. (a) The lightness of the contour is black. (b) The lightness of the contour is 45% grey. (c) The lightness of the contour is 55% grey. (d) The lightness of the contour is white.

Also, the induced colour homogeneously spreads to the inside of the contour of the noncoloured object(s). This capturing of colour spreading between the contours has previously been shown in modified versions of the Hermann grid illusion (Jung, 1973; Spillmann, 1994; Vergeer & van Lier, 2010). In the classic version of the Hermann grid illusion, the blurred dark spot appears in the area of intersection of the crossing light bars when the background was dark (Figure 4(a)). The blob disappears when it is observed by the foveal vision. When the intersection is outlined by a contour, the brightness of the blob spreads homogeneously within the outlined area (Figure 4(b)). Under these conditions, the illusory colour survives in the foveal vision. Moreover, when the surrounding squares are coloured, the illusory blobs also become coloured (Figure 4(c); Oehler & Spillmann, 1981; Schiller & Carvey, 2005). Then, the homogenising and capturing effect around the outlines extends not only to the lightness (contrast) domain but also to the colour (hue) domain (Figure 4(d); van Lier, 2017; Vergeer & van Lier, 2010). Furthermore, the spreading colours in some of the other filling-in effects (i.e., the neon-like spreading, the watercolour effect, and colour filling-in after effect) have the same “homogeneity.” According to these common points, it plausible to theorise that these phenomena have a common mechanism.

**Figure 4.**
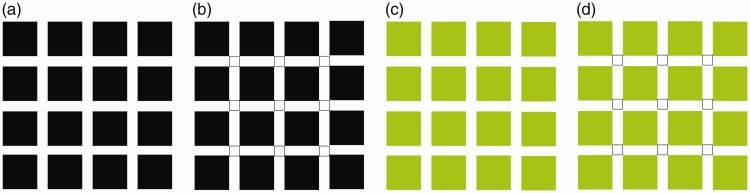
The Hermann grid illusion. (a) The classic version, (b) the contour capturing version, (c) the coloured version of the Hermann grid illusion, and (d) the contour capturing version of the expectation for hue domain.

The contour capturing version of the Hermann grid illusion and the present illusion both occur in the foveal vision, whereas this is not the case for the classic version of the Hermann grid illusion. The disappearance in the classic version of the Hermann grid illusion in the fovea is consistent with the lateral inhibition account (Baumgartner, 1960), the fact that receptive fields in the fovea are smaller than receptive fields in the peripheral area of the retina. Just like this, the classic version of the Hermann grid illusion disappears when the white bars are extremely wide (Figure 5(a)), but the contour capturing survives (Figure 5(b) and (c)). This difference suggests that the contour capturing is independent of the size of the receptive fields of the retinal ganglion cell. That is, these findings point to the need for another mechanism to explain the contour capturing illusory colour in addition to the lateral inhibition account.

**Figure 5.**
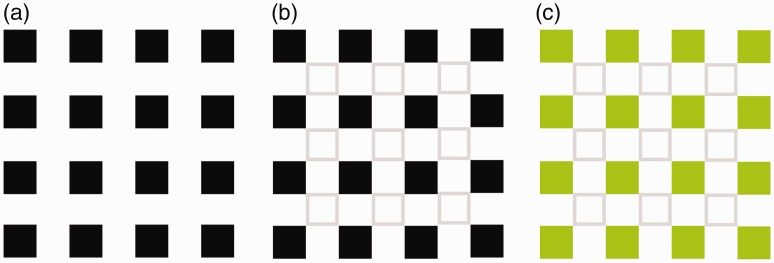
The classic (a) and the contour capturing versions of Hermann grid pattern (b and c) with extreme wide white bars.

Based upon our informal observation, the present illusion has two characteristic aspects: the presence of a “gap” and the contour capturing. To obtain a more objective perspective of this illusion, we conducted a preliminary experiment to investigate whether naïve individuals would report an illusory colour propagating into the inner square of a pair of concentric squares. Furthermore, given the results of the preliminary experiment, two additional experiments were conducted.

## Preliminary Experiment and Experiment 1

### Methods

#### Participants

Three naïve observers (DH, RK, and YK) participated in the preliminary experiment, and 12 naïve observers participated in Experiment 1. All participants reported having normal or corrected-to-normal vision, and they were not familiar with the purpose of the experiment. This study was approved by the review board of the National Institute of Advanced Industrial Science and Technology. All participants provided written informed consent and gave permission for their data to be used in the analysis.

#### Apparatus and stimuli

The stimuli were generated using MATLAB (Math Works) and the Psychophysics Toolbox (Brainard, 1997) and were controlled by a computer (Apple iMac 2015). The screen refresh rate was 60 Hz. The monitor was calibrated using the X-Rite monitor optimiser. The viewing distance was 90 cm.

**Figure 6.**
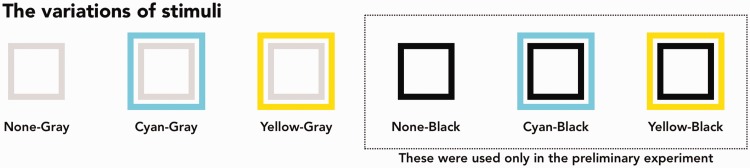
The variations of stimuli in the present study.

Six variations of the pair of concentric squares were the stimuli (Figure 6). There were three variations of the outer square: none, cyan (CIE (x, y) = 0.2247, 0.3288; 78.17 cd/m^2^) and yellow (CIE (x, y) = 0.4193, 0.5052; 95.20 cd/m^2^); and two variations of the inner square: grey (CIE (x, y) = 0.3127, 0.3290; 74.51 cd/m^2^) and black (CIE (x, y) = 0.3127, 0.3290; 12.50 cd/m^2^). All pairs were used in the preliminary experiment, and three pairs (the combinations of three variations of the outer square and a grey inner square) were used in Experiment 1. The background was white (CIE (x, y) = 0.3127, 0.3290; 99.9 cd/m^2^). The length of a side of the outer and inner squares was 2.1° and 1.4°, respectively. The line weight was 0.2° for both outer and inner squares.

#### Procedure

**Figure 7.**
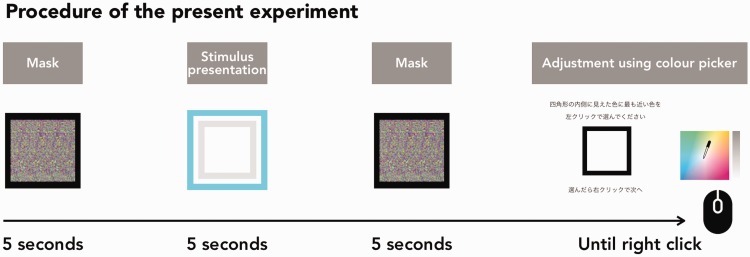
The sequence of a trial in the preliminary experiment and Experiment 1.

First, a 5-second random flashed mask (RGB values were randomly changed within the range of 128 ± 64 in each dot and were redrawn every time the screen refreshed) was presented. Then, a pair of concentric squares was presented for 5 seconds. Next, a 5-second random flashed mask was presented again to reject an aftereffect. Finally, the single square (the length of a side was 1.4°, line weight was 0.2°, and line colour was black) for the presentation of the adjusted colour and the in-house colour picker were presented. The in-house colour picker had an equiluminant plane of CIE xy colour space for the hue adjustment and a grey-scale bar for the brightness adjustment; it was presented on the left side 10° away from the centre of the screen. Then, the observers were required to recall and reproduce the perceived colour inside of the inner square of the previous concentric squares using the colour picker. The colour picker allowed the observer to adjust the colour by a left-click in the colour space, and the brightness was adjusted by a left-click in the vertical grey-scale bar. If the observers clicked anywhere, the provisional adjusted colour was presented inside of the single square in the centre of the screen. There was no time limit for this adjustment task. When the observer determined that the perceived colour was reproduced, they would then right-click to progress to the next trial. If the observers concluded that the perceived colour inside of the inner square and background was the same when they were observing the stimulus (i.e., the observers concluded that the perceived colour inside of the inner square was white), they would right-click without left-click anywhere (Figure 7). Each stimulus was presented 4 times in the preliminary experiment and 10 times in Experiment 1. The trial sequence was randomised in both experiments.

### Results and Discussion

**Figure 8.**
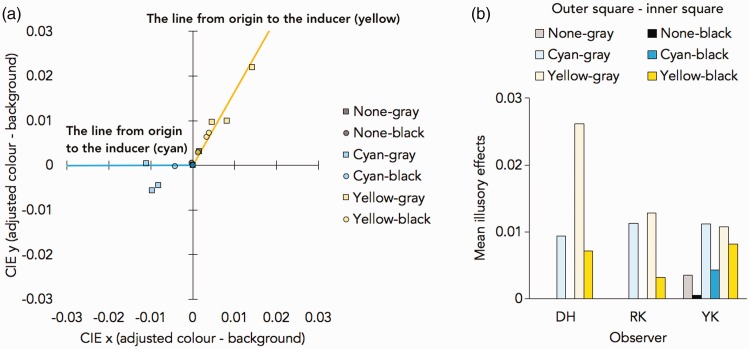
The results of preliminary experiment. (a) The summary of the adjusted colour and the mean CIE (x, y) colour value (difference from the background; x = 0.3127, y = 0.3290). The lines from origin to the inducer (the colour of the contour of the outer square) are drawn in yellow and cyan. (b) The illusory effect (the Euclidean distances between the adjusted and the background CIE colour values) in three observers of the preliminary experiment.

**Figure 9.**
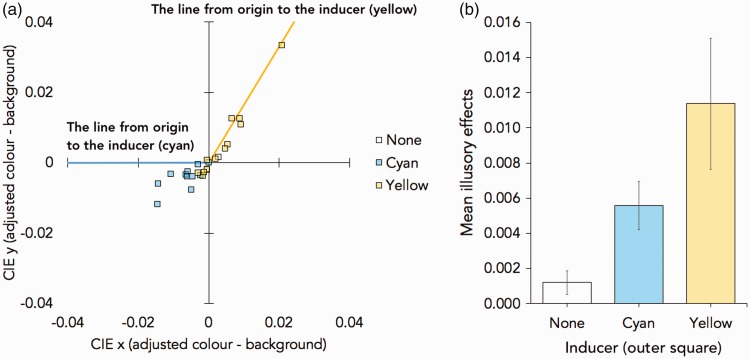
The results in Experiment 1. (a) The summary of the adjusted colour and the mean CIE (x, y) colour value (difference from the background; x = 0.3127, y = 0.3290). The lines from origin to the inducer (the colour of the contour of the outer square) are drawn in yellow and cyan. (b) Mean illusory effects (the Euclidean distances between the adjusted and the background CIE colour values). Error bars show one standard error of the mean.

Figure 8(a) shows the adjusted CIE colour values (x, y). The results showed that the illusory colour tended to depend on the colour of the outer square, and the illusory colour was stronger when the inner square was lightly coloured rather than darkly outlined. For the evaluation of the illusory effect, we calculated the Euclidean distances between the adjusted and the background CIE colour values as the magnitudes of illusory colour in the preliminary experiment (Figure 8(b)) and Experiment 1 (Figure 9).

Direct comparisons between the illusory effect in each condition of Experiment 1 and background (value = 0) using one-sample *t* tests were conducted. There were the significant effects when the outer square colour was cyan, *t*(11) = 4.259, *p* = .001, Cohen’s *d* = 1.229; and yellow, *t*(13) = 3.186, *p* = .009, Cohen’s *d* = 0.920. There was no significant effect when the outer square was not presented, *t*(13) = 1.859, *p* = .090, Cohen’s *d* = 0.537. Next, we conducted a one-way repeated-measures analysis of variance (ANOVA) with the outer square conditions. There was a main effect of the outer square conditions, *F*(1.217, 13.385) = 6.803, *p* = .017, η^2 ^= 0.382 (the sphericity corrections using Greenhouse-Geisser method were conducted because the violation of sphericity assumption was detected in Mauchly’s test, *W* = .356, *p* = .006, Greenhouse-Geisser ɛ = .608). Post hoc analysis using Holm’s method showed that the illusory colour was stronger when the outer coloured square was present than when it was absent; cyan (*p* = .048, Cohen’s *d* = 0.819) and yellow (*p* = .048, Cohen’s *d* = 0.813).

In the preliminary experiment and Experiment 1, the illusory colour was reported as being the same hue as the nearby coloured object. Furthermore, the illusory colour was stronger when the contour was grey than when black. All results were consistent with our informal observations.

## Experiment 2

In Experiment 2, the effects of presentation time were examined. As mentioned earlier, the illusory colour may grow progressively stronger when an observer stares at an image for a longer period of time. Thus, we manipulated the presentation time of the stimulus to be between 0.1 and 5 seconds. At the same time, the reproducibility of the present phenomenon was tested.

### Methods

Fourteen naïve observers participated in Experiment 2. All participants reported normal or corrected-to-normal vision and were not familiar with the purpose of the experiment. This study was approved by the review board of the National Institute of Advanced Industrial Science and Technology. All participants provided written informed consent and gave permission for their data to be used in the analysis.

Apparatus, the stimuli, experimental design, and the procedure were the same as in Experiment 1 with the following exception. Only one variation of the pair of concentric squares (yellow–grey pair) was used in Experiment 2. There were five variations of the presentation time condition for the stimulus: 0.1, 0.5, 1, 3, or 5 seconds.

### Results and Discussion

**Figure 10.**
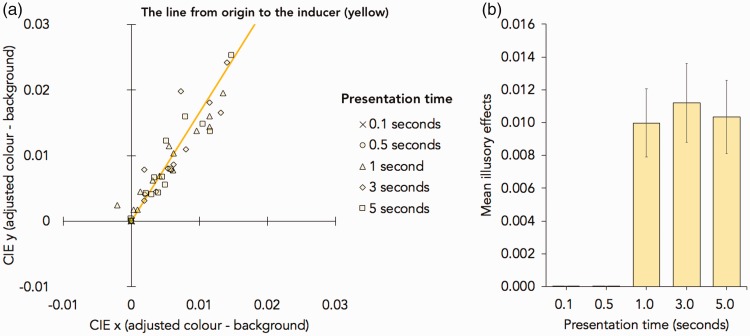
The results in Experiment 2. (a) The summary of the adjusted colour and the mean CIE (x, y) colour value (difference from the background; x = 0.3127, y = 0.3290). The line from origin to the inducer (the colour of the contour of the outer square) is drawn in yellow. (b) Mean illusory effects (the Euclidean distances between the adjusted and the background CIE colour values). Error bars show one standard error of the mean.

The illusory effects in each presentation time condition were calculated in the same manner as Experiment 1, and the results are shown in Figure 10.

In the statistical analysis, the data in the short presentation conditions (0.1 or 0.5 seconds) were excluded from the analysis because none of the observers reported any illusory colour in these conditions. First, to test the direct comparisons between the illusory effect in each condition (1, 3, or 5 seconds) and background (value = 0), one-sample *t* tests were computed. There were the significant effects when the presentation time were 1 second, *t*(13) = 4.975, *p* < .001, Cohen’s *d* = 1.330; 3 seconds, *t*(13) = 4.822, *p* < .001, Cohen’s *d* = 1.289; and 5 seconds, *t*(13) = 4.975, *p* < .001, Cohen’s *d* = 1.281. Furthermore, a repeated-measures ANOVA with the presentation times (1, 3, or 5 seconds) was performed to test these effects, which showed that there was no main effect of the presentation time, *F*(2, 26) = 0.248, *p* = .782, η^2 ^= 0.019.

The illusory colour effect was also identified in Experiment 2, and it was approximately the same as the effect found in Experiment 1. Furthermore, the results showed that the illusory colour appeared after at least 0.5 seconds of stimulus onset.

## General Discussion

The present study revealed a new type of colour spreading illusion based on our informal observations and the experimental results. According to our informal observations, seven main findings were reported: (a) The illusory colour spreading occurs even if the coloured area and the colour spreading area (that is not actually coloured) are separated by a gap; (b) the illusory colour grows progressively stronger when one has been staring at the image for a while; (c) there is a good deal of flexibility in the geometric features for the appearance of the present effect; (d) the illusory colour becomes weak or disappears when the lightness contrast between the contour and the background is too high; (e) the brightness (whiteness) of the gap (i.e., the area between coloured area and the achromatic contour) appears to be enhanced compared with the background at the same time that illusory colour appears; (f) the illusory colour homogeneously spreads to the inside of the contour; and (g) the illusory colour survives in the foveal vision.

These findings suggest that the present illusory colour spreading has some similar features to the contour capturing of colour such as the modified version of the Hermann grid illusion (Vergeer & van Lier, 2010). To fully explain the mechanism of the present illusory colour spreading, an account that differs from the classic account of the Hermann grid illusion is needed: the account of the lateral inhibition at the retinal ganglion cell (Baumgartner, 1960).

We consider the contour-based perception account (Francis, 2010) to be a reasonable explanation for the present effect. In their pioneering studies, Grossberg and Mingolla (1985a, 1985b) proposed the account of the contour-based visual system. This account argued that two complementary systems are subsystems of the object recognition system: the feature contour system and the boundary contour system. First, a monocular preprocessed signal is sent to both systems. The boundary contour system generates the boundary structures by edge detection, and the feature contour system represents the information of surface properties such as luminance and hue within a scene. Then, the representations of the two systems were coherently integrated. The contour capturing of illusory colour may reflect the integral process of information from these systems.

To further test this account, further empirical studies as well as a model simulation approach are necessary. As a first step, we performed a preliminary experiment and two full experiments to obtain a more objective perspective of our illusion in the present study. The preliminary experiment and Experiment 1 demonstrated the objective nature of the present effect. The results were consistent with our informal observations that the illusory colour was the same hue as the nearby coloured object. Furthermore, the illusory colour was stronger when the contrast between the background and the contour for the capturing illusory colour was low rather than high.

In Experiment 2, the results of Experiment 1 were completely reproduced, and it further showed that the illusory colour appears after at least 0.5 seconds from the stimulus onset. This suggests that the contour capturing of illusory colour may reflect the later stage of visual processes, such as the cortical process or the slow processing at the retina. One possible account for the present findings is that an illusory colour is caused by a combination of some different components of surround suppression at the cortical level. Recent studies have suggested that the mechanism of surround suppression may be divided into different components in the visual cortex (Bair, Cavanaugh, & Movshon, 2003; Kilpeläinen, Donner, & Laurinen, 2007; Webb, 2005); however, the evidence from the present study may be insufficient for the verification of this account. Verification of this mechanism will be examined in future research.

In summary, the present study showed a new type of colour spreading illusion. It occurs even if the coloured area and the colour spreading area are separated. One possible explanation of the present illusion—the contour-based perception account and the relationship with surround suppression at the cortical level—was discussed. Although the mechanisms underlying this effect remain unclear, the present findings have interesting implications for our understanding of colour perception.
